# Supplementary material for: New Transition Metal Coordination Polymers Derived from 2-(3,5-Dicarboxyphenyl)-6-carboxybenzimidazole as Photocatalysts for Dye and Antibiotic Decomposition
Source: Molecules. 2023 Oct 28;28(21):7318. doi: 10.3390/molecules28217318 (PMC10648955; doi:10.3390/molecules28217318)

checkCIF/PLATON report

You have not supplied any structure factors. As a result the full set of tests cannot be run.

THIS REPORT IS FOR GUIDANCE ONLY. IF USED AS PART OF A REVIEW PROCEDURE FOR PUBLICATION, IT SHOULD NOT REPLACE THE EXPERTISE OF AN EXPERIENCED CRYSTALLOGRAPHIC REFEREE.

No syntax errors found. CIF dictionary Interpreting this report

Datablock: 1

Bond precision:	C-C = 0.0030 A		Wavelength=0.71073
Cell:	a=13.9385(10)	b=15.4139(10)	c=16.8615(12)
	alpha=90	beta=106.6609(11)	gamma=90
Temperature:	296 K		

	Calculated	Reported
Volume	3470.6(4)	3470.6(4)
Space group	C 2/c	C 1 2/c 1
Hall group	-C 2yc	-C 2yc
Moiety formula	C16 H12 N2 Ni O8 [+ solvent]	C16 H12 N2 Ni O8, 1.9[H2O]
Sum formula	C16 H12 N2 Ni O8 [+ solvent]	C16 H15.80 N2 Ni O9.90
Mr	418.97	453.22
Dx,g cm-3	1.604	1.735
Z	8	8
Mu (mm-1)	1.167	1.181
F000	1712.0	1864.0
F000'	1715.61	
h,k,lmax	18,20,21	18,19,21
Nref	4053	3937
Tmin,Tmax	0.775,0.828	0.641,0.746
Tmin'	0.718	

Correction method= # Reported T Limits: Tmin=0.641 Tmax=0.746
AbsCorr = MULTI-SCAN

Data completeness= 0.971 Theta(max)= 27.649

R(reflections)= 0.0353(3270) wR2(reflections)= 0.1048(3937)

S = 1.022 Npar= 247

The following ALERTS were generated. Each ALERT has the format
test-name_ALERT_alert-type_alert-level.
Click on the hyperlinks for more details of the test.

Alert level B

```
PLAT420_ALERT_2_B D-H Bond Without Acceptor  O8       --H8B      .     Please Check
```

Author Response: Slovent Mask was used during the refinement process, and the solvent was deducted, resulting in H8B on O8 and H2 on N2 no longer having receptors to form hydrogen bonds with it.

Alert level C

```
PLAT250_ALERT_2_C Large U3/U1 Ratio for Average U(i,j) Tensor ....        2.5 Note
PLAT420_ALERT_2_C D-H Bond Without Acceptor  N2       --H2       .     Please Check
```

Author Response: Slovent Mask was used during the refinement process, and the solvent was deducted, resulting in H8B on O8 and H2 on N2 no longer having receptors to form hydrogen bonds with it.

Alert level G

```
FORMU01_ALERT_2_G  There is a discrepancy between the atom counts in the
            _chemical_formula_sum and the formula from the _atom_site* data.
            Atom count from _chemical_formula_sum:C16 H15.8 N2 Ni1 O9.9
            Atom count from the _atom_site data:  C16 H12 N2 Ni1 O8
CELLZ01_ALERT_1_G Difference between formula and atom_site contents detected.
CELLZ01_ALERT_1_G ALERT: Large difference may be due to a
            symmetry error - see SYMMG tests
           From the CIF: _cell_formula_units_Z    8
           From the CIF: _chemical_formula_sum  C16 H15.80 N2 Ni O9.90
           TEST: Compare cell contents of formula and atom_site data

           atom    Z*formula  cif sites diff
           C        128.00    128.00    0.00
           H        126.40     96.00   30.40
           N         16.00     16.00    0.00
           Ni         8.00      8.00    0.00
           O         79.20     64.00   15.20
PLAT004_ALERT_5_G Polymeric Structure Found with Maximum Dimension          2 Info
PLAT007_ALERT_5_G Number of Unrefined Donor-H Atoms .............          6 Report
PLAT041_ALERT_1_G Calc. and Reported SumFormula    Strings  Differ     Please Check
PLAT232_ALERT_2_G Hirshfeld Test Diff (M-X)  Ni1      --O6_d     .        6.2 s.u.
PLAT605_ALERT_4_G Largest Solvent Accessible VOID in the Structure        126 A**3
```

```
PLAT794_ALERT_5_G Tentative Bond Valency for Ni1       (II)      .       2.04 Info
PLAT868_ALERT_4_G ALERTS Due to the Use of _smtbx_masks Suppressed          ! Info
PLAT941_ALERT_3_G Average HKL Measurement Multiplicity ..........        2.6 Low
```

```
   0 ALERT level A = Most likely a serious problem - resolve or explain
   1 ALERT level B = A potentially serious problem, consider carefully
   2 ALERT level C = Check. Ensure it is not caused by an omission or oversight
  11 ALERT level G = General information/check it is not something unexpected

   3 ALERT type 1 CIF construction/syntax error, inconsistent or missing data
   5 ALERT type 2 Indicator that the structure model may be wrong or deficient
   1 ALERT type 3 Indicator that the structure quality may be low
   2 ALERT type 4 Improvement, methodology, query or suggestion
   3 ALERT type 5 Informative message, check
```

It is advisable to attempt to resolve as many as possible of the alerts in all categories. Often the minor alerts point to easily fixed oversights, errors and omissions in your CIF or refinement strategy, so attention to these fine details can be worthwhile. In order to resolve some of the more serious problems it may be necessary to carry out additional measurements or structure refinements. However, the purpose of your study may justify the reported deviations and the more serious of these should normally be commented upon in the discussion or experimental section of a paper or in the "special_details" fields of the CIF. checkCIF was carefully designed to identify outliers and unusual parameters, but every test has its limitations and alerts that are not important in a particular case may appear. Conversely, the absence of alerts does not guarantee there are no aspects of the results needing attention. It is up to the individual to critically assess their own results and, if necessary, seek expert advice.

Publication of your CIF in IUCr journals

A basic structural check has been run on your CIF. These basic checks will be run on all CIFs submitted for publication in IUCr journals (*Acta Crystallographica*, *Journal of Applied Crystallography*, *Journal of Synchrotron Radiation*); however, if you intend to submit to *Acta Crystallographica Section C* or *E* or *IUCrData*, you should make sure that full publication checks are run on the final version of your CIF prior to submission.

Publication of your CIF in other journals

Please refer to the *Notes for Authors* of the relevant journal for any special instructions relating to CIF submission.

PLATON version of 06/07/2023; check.def file version of 30/06/2023

Datablock 1 - ellipsoid plot

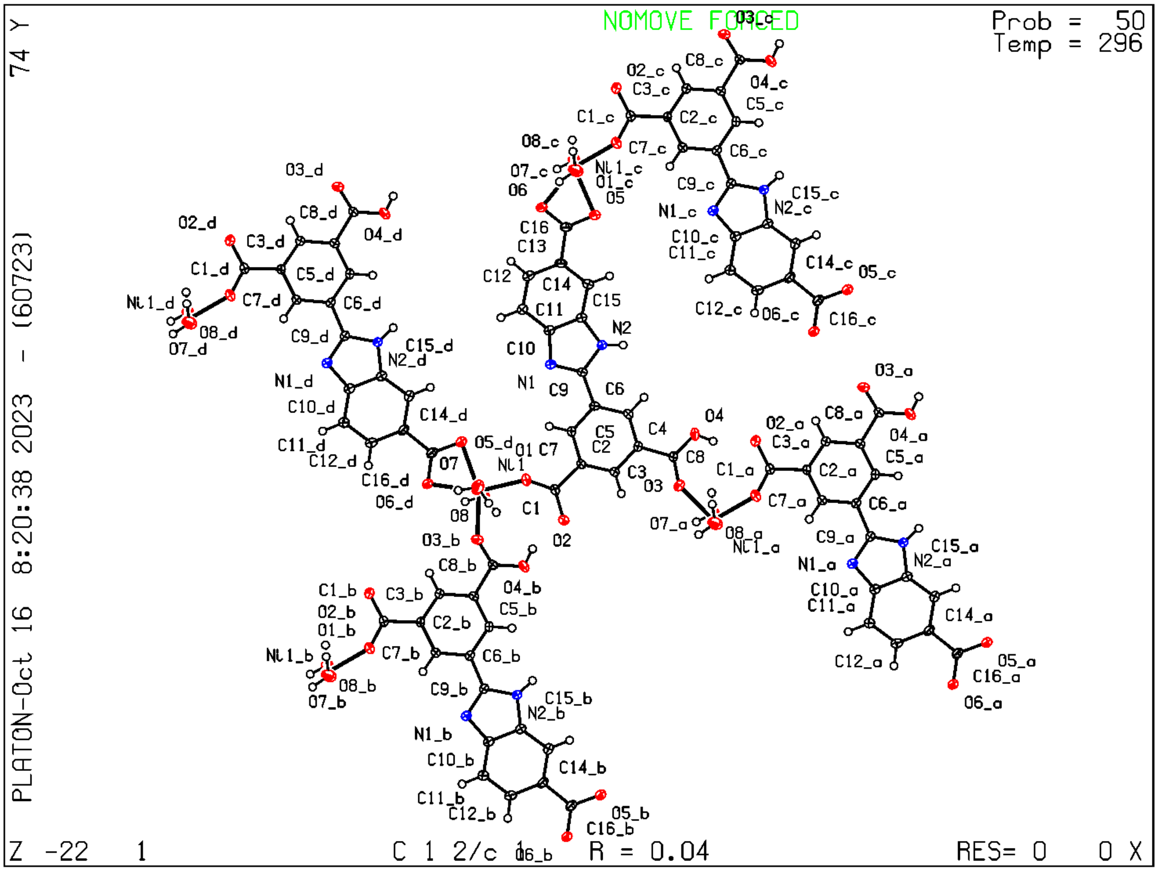

Supplement: Supplementary file 1 [file molecules-28-07318-s001.zip › complex1 checkcif.pdf]
