# Supplementary material for: New Transition Metal Coordination Polymers Derived from 2-(3,5-Dicarboxyphenyl)-6-carboxybenzimidazole as Photocatalysts for Dye and Antibiotic Decomposition
Source: Molecules. 2023 Oct 28;28(21):7318. doi: 10.3390/molecules28217318 (PMC10648955; doi:10.3390/molecules28217318)

checkCIF/PLATON report

You have not supplied any structure factors. As a result the full set of tests cannot be run.

THIS REPORT IS FOR GUIDANCE ONLY. IF USED AS PART OF A REVIEW PROCEDURE FOR PUBLICATION, IT SHOULD NOT REPLACE THE EXPERTISE OF AN EXPERIENCED CRYSTALLOGRAPHIC REFEREE.

No syntax errors found. CIF dictionary Interpreting this report

Datablock: 1

```
Bond precision:   C-C = 0.0094 A           Wavelength=0.71073

Cell:           a=11.1841(9)       b=16.4828(14)     c=16.7565(14)
                alpha=115.662(1)   beta=98.114(2)    gamma=105.482(2)
Temperature:    296 K

                 Calculated                         Reported
Volume           2563.4(4)                          2563.4(4)
Space group      P -1                               P -1
Hall group       -P 1                               -P 1
Moiety formula   C56 H33 Mn3 N8 O14, 2(H2 O) C56 H33 Mn3 N8 O14, 2(H2 O)
Sum formula      C56 H37 Mn3 N8 O16                 C56 H37 Mn3 N8 O16
Mr               1242.76                            1242.75
Dx,g cm-3        1.610                              1.610
Z                2                                  2
Mu (mm-1)        0.812                              0.812
F000             1264.0                             1264.0
F000'            1266.59
h,k,lmax         14,21,21                           14,21,21
Nref             11795                              11158
Tmin,Tmax        0.839,0.878                        0.672,0.746
Tmin'            0.810

Correction method= # Reported T Limits: Tmin=0.672 Tmax=0.746
AbsCorr = MULTI-SCAN

Data completeness= 0.946            Theta(max)= 27.512

                                                     wR2(reflections)=
R(reflections)= 0.0654( 6623)                        0.2047( 11158)

S = 1.065                    Npar= 776
```

The following ALERTS were generated. Each ALERT has the format
test-name_ALERT_alert-type_alert-level.
Click on the hyperlinks for more details of the test.

Alert level C

```
PLAT094_ALERT_2_C Ratio of Maximum / Minimum Residual Density ....       2.28 Report
PLAT220_ALERT_2_C NonSolvent   Resd 1  C   Ueq(max)/Ueq(min) Range        3.3 Ratio
PLAT220_ALERT_2_C NonSolvent   Resd 1  O   Ueq(max)/Ueq(min) Range        4.2 Ratio
PLAT222_ALERT_3_C NonSolvent Resd 1  H   Uiso(max)/Uiso(min) Range        5.7 Ratio
PLAT241_ALERT_2_C High   'MainMol' Ueq as Compared to Neighbors of        C37 Check
PLAT242_ALERT_2_C Low    'MainMol' Ueq as Compared to Neighbors of        Mn1 Check
PLAT341_ALERT_3_C Low Bond Precision on  C-C Bonds ..............      0.0094 Ang.
PLAT369_ALERT_2_C Long   C(sp2)-C(sp2) Bond  C20      - C24      .       1.53 Ang.
PLAT411_ALERT_2_C Short Inter H...H Contact  H3       ..H30      .       2.12 Ang.
                                                 2-x,-y,1-z  =      2_756 Check
PLAT417_ALERT_2_C Short Inter D-H..H-D       H13      ..H15B     .       2.11 Ang.
                                                      x,y,z  =      1_555 Check
PLAT601_ALERT_2_C Unit Cell Contains Solvent Accessible VOIDS of .         75 Ang**3
```

Alert level G

```
PLAT002_ALERT_2_G Number of Distance or Angle Restraints on AtSite          9 Note
PLAT003_ALERT_2_G Number of Uiso or Uij Restrained non-H Atoms ...         13 Report
PLAT004_ALERT_5_G Polymeric Structure Found with Maximum Dimension          2 Info
PLAT007_ALERT_5_G Number of Unrefined Donor-H Atoms .............          10 Report
PLAT172_ALERT_4_G The CIF-Embedded .res File Contains DFIX Records          4 Report
PLAT174_ALERT_4_G The CIF-Embedded .res File Contains FLAT Records          2 Report
PLAT176_ALERT_4_G The CIF-Embedded .res File Contains SADI Records          1 Report
PLAT177_ALERT_4_G The CIF-Embedded .res File Contains DELU Records          1 Report
PLAT178_ALERT_4_G The CIF-Embedded .res File Contains SIMU Records          1 Report
PLAT186_ALERT_4_G The CIF-Embedded .res File Contains ISOR Records          2 Report
PLAT188_ALERT_3_G A Non-default SIMU Restraint Value has been used     0.0100 Report
PLAT191_ALERT_3_G A Non-default SADI Restraint Value has been used     0.0100 Report
PLAT301_ALERT_3_G Main Residue  Disorder .............(Resd  1  )          4% Note
PLAT480_ALERT_4_G Long H...A H-Bond Reported H34      ..O2       .       2.63 Ang.
PLAT794_ALERT_5_G Tentative Bond Valency for Mn1       (II)      .       2.07 Info
PLAT794_ALERT_5_G Tentative Bond Valency for Mn2       (II)      .       1.94 Info
PLAT794_ALERT_5_G Tentative Bond Valency for Mn3       (II)      .       2.02 Info
PLAT860_ALERT_3_G Number of Least-Squares Restraints ............         200 Note
PLAT883_ALERT_1_G No Info/Value for _atom_sites_solution_primary .     Please Do !
PLAT941_ALERT_3_G Average HKL Measurement Multiplicity ..........         1.4 Low
```

```
   0 ALERT level A = Most likely a serious problem - resolve or explain
   0 ALERT level B = A potentially serious problem, consider carefully
  11 ALERT level C = Check. Ensure it is not caused by an omission or oversight
  20 ALERT level G = General information/check it is not something unexpected

   1 ALERT type 1 CIF construction/syntax error, inconsistent or missing data
  11 ALERT type 2 Indicator that the structure model may be wrong or deficient
   7 ALERT type 3 Indicator that the structure quality may be low
   7 ALERT type 4 Improvement, methodology, query or suggestion
   5 ALERT type 5 Informative message, check
```

It is advisable to attempt to resolve as many as possible of the alerts in all categories. Often the minor alerts point to easily fixed oversights, errors and omissions in your CIF or refinement strategy, so attention to these fine details can be worthwhile. In order to resolve some of the more serious problems it may be necessary to carry out additional measurements or structure refinements. However, the purpose of your study may justify the reported deviations and the more serious of these should normally be commented upon in the discussion or experimental section of a paper or in the "special_details" fields of the CIF. checkCIF was carefully designed to identify outliers and unusual parameters, but every test has its limitations and alerts that are not important in a particular case may appear. Conversely, the absence of alerts does not guarantee there are no aspects of the results needing attention. It is up to the individual to critically assess their own results and, if necessary, seek expert advice.

Publication of your CIF in IUCr journals

A basic structural check has been run on your CIF. These basic checks will be run on all CIFs submitted for publication in IUCr journals (*Acta Crystallographica*, *Journal of Applied Crystallography*, *Journal of Synchrotron Radiation*); however, if you intend to submit to *Acta Crystallographica Section C* or *E* or *IUCrData*, you should make sure that full publication checks are run on the final version of your CIF prior to submission.

Publication of your CIF in other journals

Please refer to the *Notes for Authors* of the relevant journal for any special instructions relating to CIF submission.

PLATON version of 06/07/2023; check.def file version of 30/06/2023

Datablock 1 - ellipsoid plot

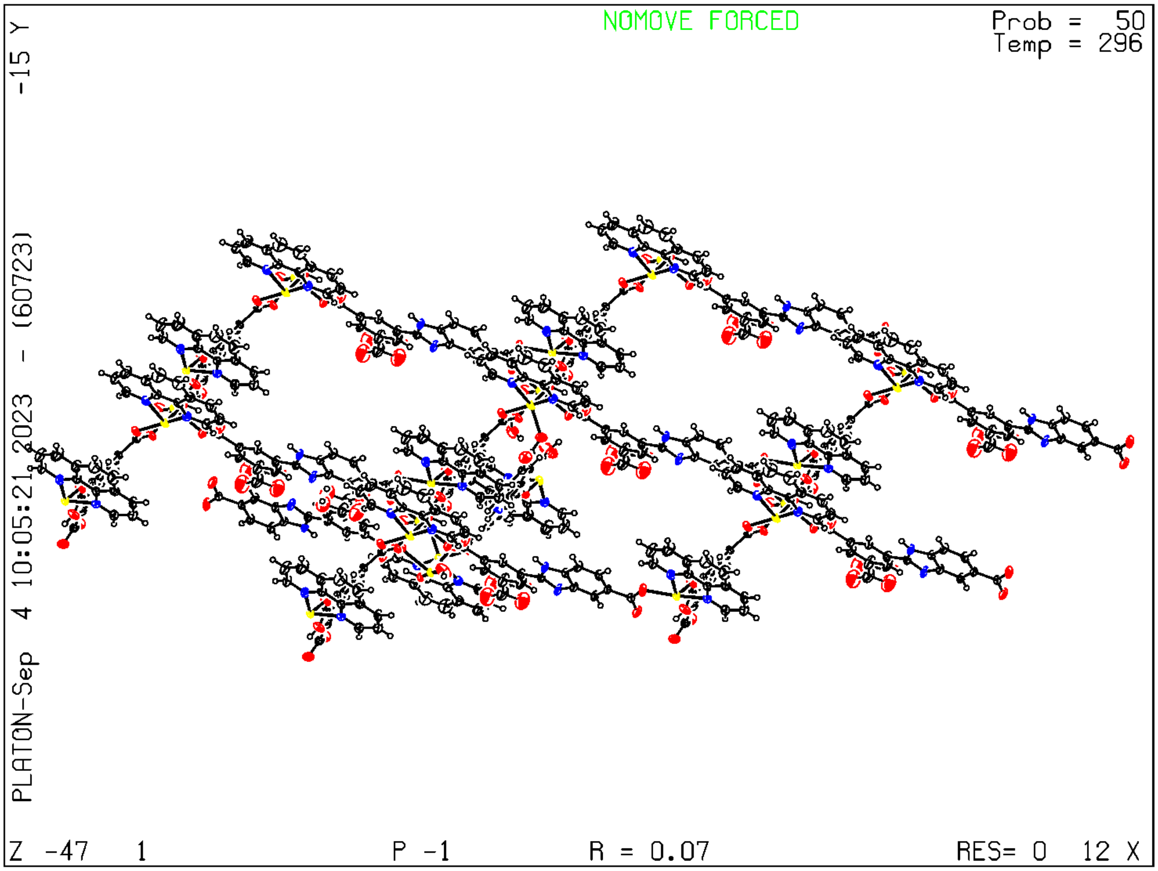

Supplement: Supplementary file 1 [file molecules-28-07318-s001.zip › complex2 checkcif.pdf]
