# Supplementary material for: New Transition Metal Coordination Polymers Derived from 2-(3,5-Dicarboxyphenyl)-6-carboxybenzimidazole as Photocatalysts for Dye and Antibiotic Decomposition
Source: Molecules. 2023 Oct 28;28(21):7318. doi: 10.3390/molecules28217318 (PMC10648955; doi:10.3390/molecules28217318)

checkCIF/PLATON report

You have not supplied any structure factors. As a result the full set of tests cannot be run.

THIS REPORT IS FOR GUIDANCE ONLY. IF USED AS PART OF A REVIEW PROCEDURE FOR PUBLICATION, IT SHOULD NOT REPLACE THE EXPERTISE OF AN EXPERIENCED CRYSTALLOGRAPHIC REFEREE.

No syntax errors found. CIF dictionary Interpreting this report

Datablock: wy-4_auto_sq

Bond precision:	C-C = 0.0160 A		Wavelength=0.71073
Cell:	a=13.4259(17)	b=15.3909(15)	c=15.619(2)
	alpha=90	beta=99.496(14)	gamma=90
Temperature:	293 K		

	Calculated	Reported
Volume	3183.2(7)	3183.2(7)
Space group	C 2/c	C 1 2/c 1
Hall group	-C 2yc	-C 2yc
Moiety formula	C16 H10 Cd N2 O7, H2 O [+ solvent]	C16 H10 Cd N2 O7, H2 O
Sum formula	C16 H12 Cd N2 O8 [+ solvent]	C16 H12 Cd N2 O8
Mr	472.69	472.68
Dx,g cm-3	1.973	1.973
Z	8	8
Mu (mm-1)	1.425	1.425
F000	1872.0	1872.0
F000'	1866.53	
h,k,lmax	18,21,21	18,21,21
Nref	4319	3895
Tmin,Tmax	0.659,0.652	0.918,1.000
Tmin'	0.646	

Correction method= # Reported T Limits: Tmin=0.918 Tmax=1.000
AbsCorr = MULTI-SCAN

Data completeness= 0.902 Theta(max)= 29.224

R(reflections)= 0.0986(1452)　　　wR2(reflections)= 0.2271(3895)

S = 0.998　　　Npar= 248

The following ALERTS were generated. Each ALERT has the format
test-name_ALERT_alert-type_alert-level.
Click on the hyperlinks for more details of the test.

Alert level B

RINTA01_ALERT_3_B The value of Rint is greater than 0.18
Rint given 0.238

Author Response: We have used long exposure time, and best crystal to obtain best data set.

PLAT020_ALERT_3_B The Value of Rint is Greater Than 0.12 .... 0.238 Report

Author Response: We have used long exposure time, and best crystal to obtain best data set.

PLAT026_ALERT_3_B Ratio Observed / Unique Reflections (too) Low .. 37% Check

Author Response: We have used long exposure time, and best crystal to obtain best data set.

PLAT420_ALERT_2_B D-H Bond Without Acceptor O4 --H4 . Please Check

Author Response: The disordered solvent molecules have been removed with the SQUEEZE routine of PLATON. So it is difficult to determine the H-bond pairs.

PLAT420_ALERT_2_B D-H Bond Without Acceptor O8 --H8A . Please Check

Author Response: The disordered solvent molecules have been removed with the SQUEEZE routine of PLATON. So it is difficult to determine the H-bond pairs.

Alert level C

PLAT234_ALERT_4_C Large Hirshfeld Difference O5 --C16 . 0.20 Ang.
PLAT260_ALERT_2_C Large Average Ueq of Residue Including O8 0.113 Check
PLAT342_ALERT_3_C Low Bond Precision on C-C Bonds .... 0.016 Ang.
PLAT415_ALERT_2_C Short Inter D-H..H-X H4 ..H14 . 2.10 Ang.
3/2-x,1/2-y,2-z = 7_657 Check

Alert level G

PLAT003_ALERT_2_G Number of Uiso or Uij Restrained non-H Atoms ... 12 Report

```
PLAT004_ALERT_5_G Polymeric Structure Found with Maximum Dimension          2 Info
PLAT007_ALERT_5_G Number of Unrefined Donor-H Atoms .............           6 Report
PLAT177_ALERT_4_G The CIF-Embedded .res File Contains DELU Records          1 Report
PLAT178_ALERT_4_G The CIF-Embedded .res File Contains SIMU Records          1 Report
PLAT199_ALERT_1_G Reported _cell_measurement_temperature ..... (K)        293 Check
PLAT200_ALERT_1_G Reported   _diffrn_ambient_temperature ..... (K)        293 Check
PLAT232_ALERT_2_G Hirshfeld Test Diff (M-X)  Cd1      --O3_a     .        5.2 s.u.
PLAT605_ALERT_4_G Largest Solvent Accessible VOID in the Structure         24 A**3
PLAT794_ALERT_5_G Tentative Bond Valency for Cd1       (II)      .       2.16 Info
PLAT860_ALERT_3_G Number of Least-Squares Restraints ............         100 Note
PLAT869_ALERT_4_G ALERTS Related to the Use of SQUEEZE  Suppressed          ! Info
PLAT883_ALERT_1_G No Info/Value for _atom_sites_solution_primary .     Please Do !
PLAT941_ALERT_3_G Average HKL Measurement Multiplicity ..........         4.5 Low
```

---

   0 **ALERT level A** = Most likely a serious problem - resolve or explain
   5 **ALERT level B** = A potentially serious problem, consider carefully
   4 **ALERT level C** = Check. Ensure it is not caused by an omission or oversight
  14 **ALERT level G** = General information/check it is not something unexpected

   3 ALERT type 1 CIF construction/syntax error, inconsistent or missing data
   6 ALERT type 2 Indicator that the structure model may be wrong or deficient
   6 ALERT type 3 Indicator that the structure quality may be low
   5 ALERT type 4 Improvement, methodology, query or suggestion
   3 ALERT type 5 Informative message, check

It is advisable to attempt to resolve as many as possible of the alerts in all categories. Often the minor alerts point to easily fixed oversights, errors and omissions in your CIF or refinement strategy, so attention to these fine details can be worthwhile. In order to resolve some of the more serious problems it may be necessary to carry out additional measurements or structure refinements. However, the purpose of your study may justify the reported deviations and the more serious of these should normally be commented upon in the discussion or experimental section of a paper or in the "special_details" fields of the CIF. checkCIF was carefully designed to identify outliers and unusual parameters, but every test has its limitations and alerts that are not important in a particular case may appear. Conversely, the absence of alerts does not guarantee there are no aspects of the results needing attention. It is up to the individual to critically assess their own results and, if necessary, seek expert advice.

Publication of your CIF in IUCr journals

A basic structural check has been run on your CIF. These basic checks will be run on all CIFs submitted for publication in IUCr journals (*Acta Crystallographica*, *Journal of Applied Crystallography*, *Journal of Synchrotron Radiation*); however, if you intend to submit to *Acta Crystallographica Section C* or *E* or *IUCrData*, you should make sure that full publication checks are run on the final version of your CIF prior to submission.

Publication of your CIF in other journals

Please refer to the *Notes for Authors* of the relevant journal for any special instructions relating to CIF submission.

PLATON version of 18/05/2022; check.def file version of 17/05/2022

Datablock wy-4_auto_sq - ellipsoid plot

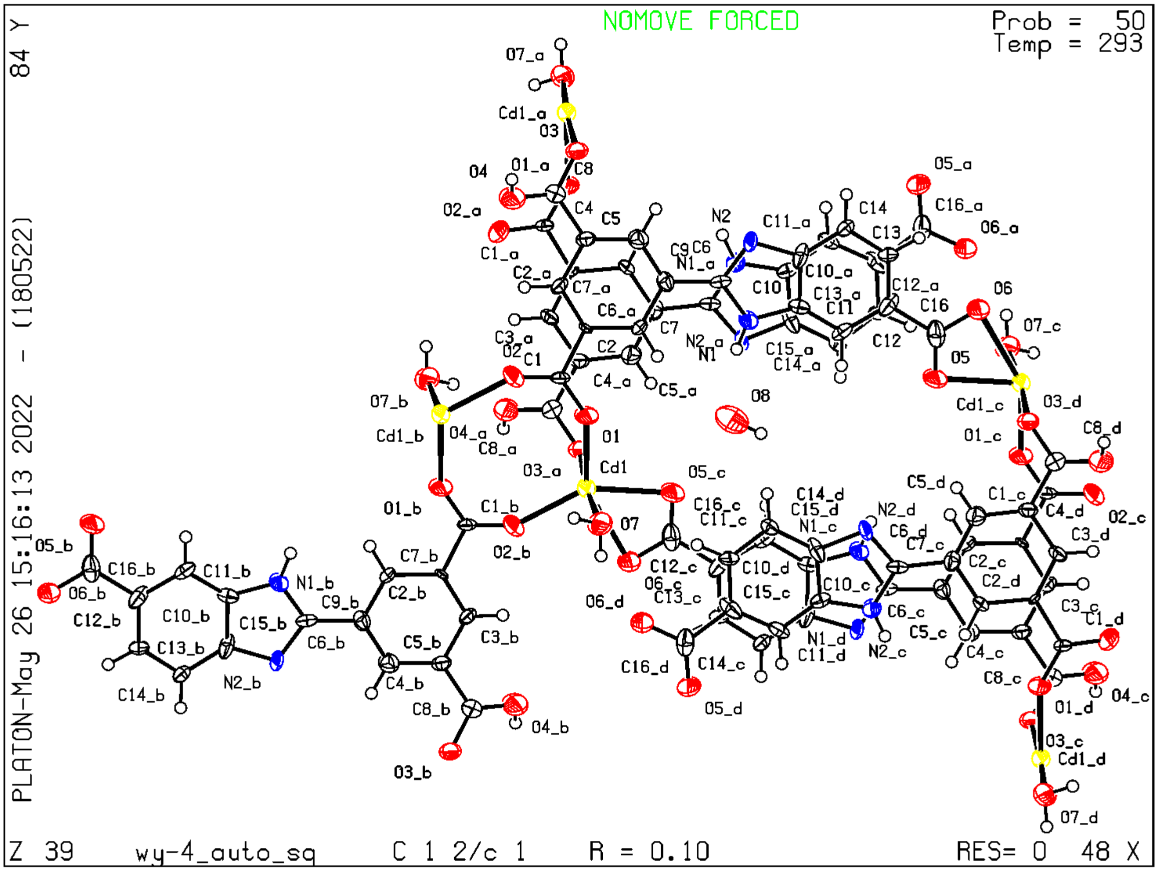

Supplement: Supplementary file 1 [file molecules-28-07318-s001.zip › complex3 checkcif.pdf]
