# Supplementary material for: New Transition Metal Coordination Polymers Derived from 2-(3,5-Dicarboxyphenyl)-6-carboxybenzimidazole as Photocatalysts for Dye and Antibiotic Decomposition
Source: Molecules. 2023 Oct 28;28(21):7318. doi: 10.3390/molecules28217318 (PMC10648955; doi:10.3390/molecules28217318)
Supplement: Supplementary file 1 [file molecules-28-07318-s001.zip › molecules-2694571-supplementary.pdf]

Supporting Information

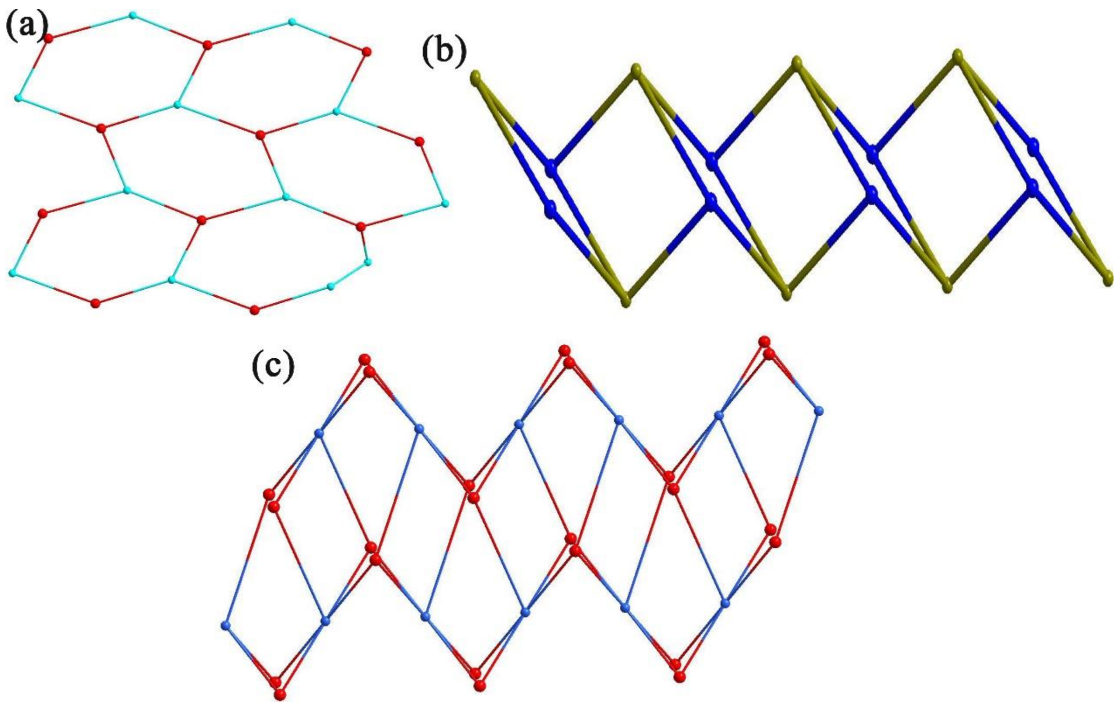

Figure. S1 different topological structures of CPs **1-3**.

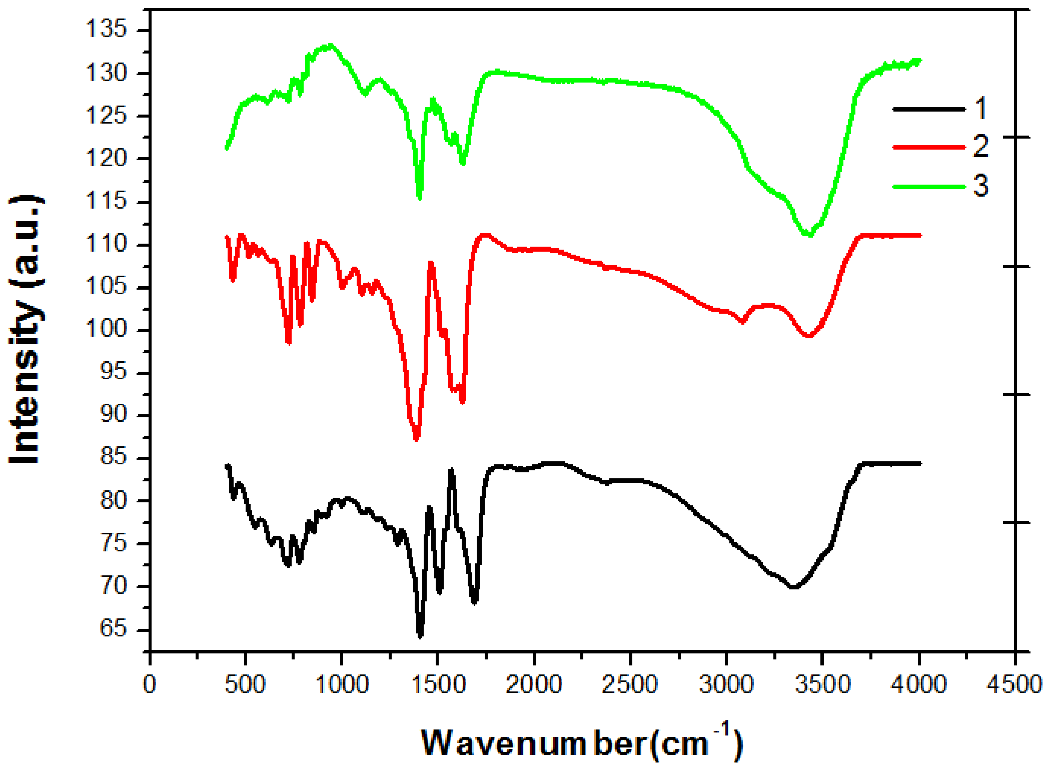

Figure. S2 view of the IR spectra of CPs **1-3**.

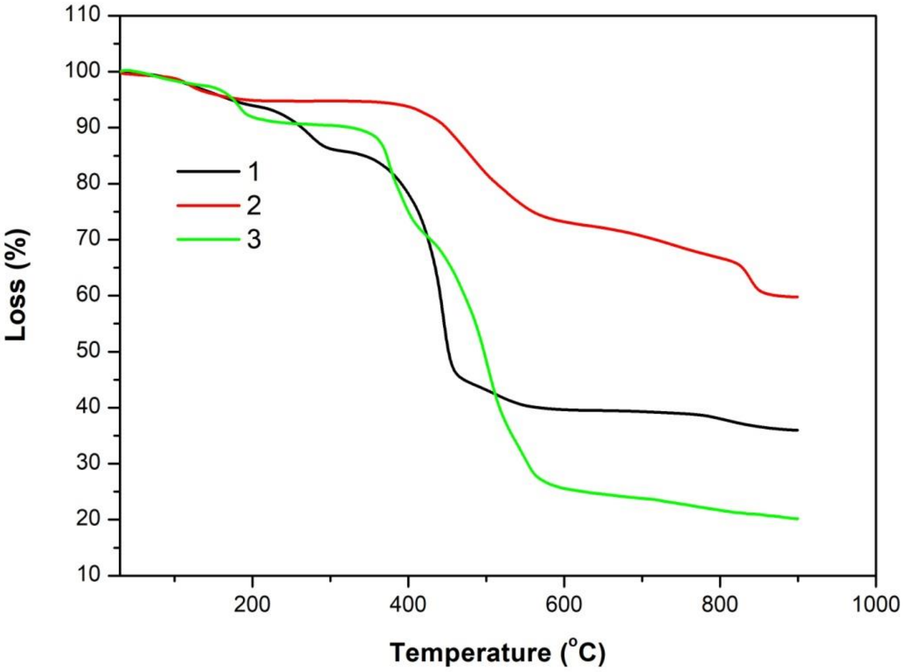

Figure. S3 The TGA profiles of CPs.

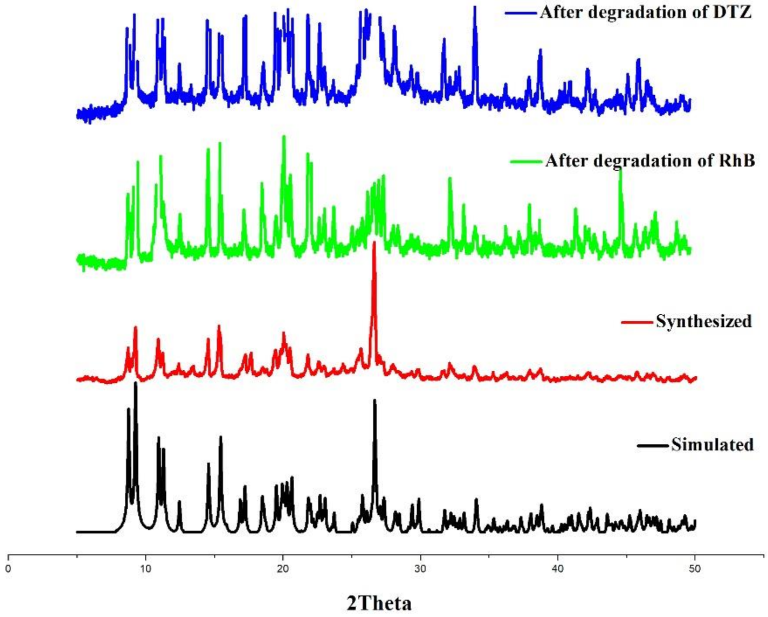

Figure. S4 PXRD plots for CP **1**.

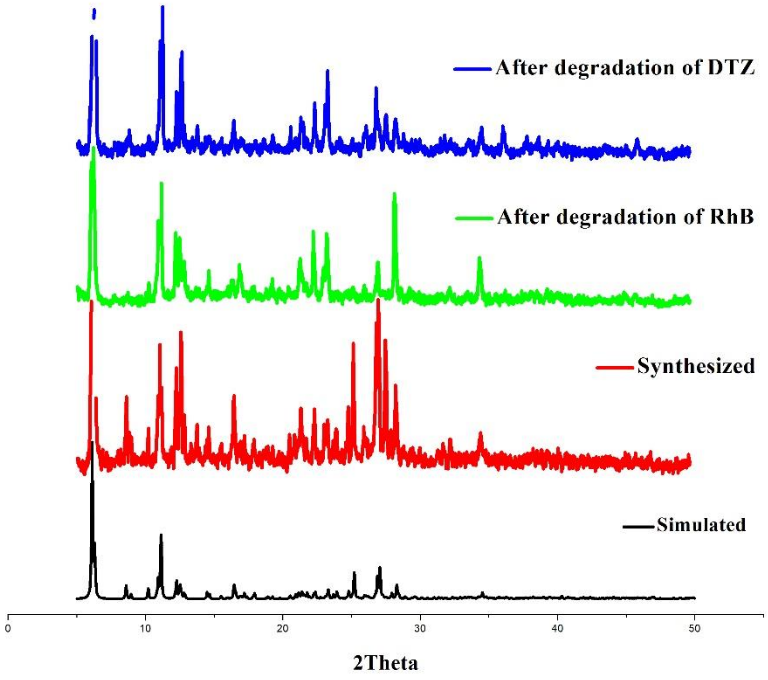

Figure. S5 PXRD plots for CP **2**.

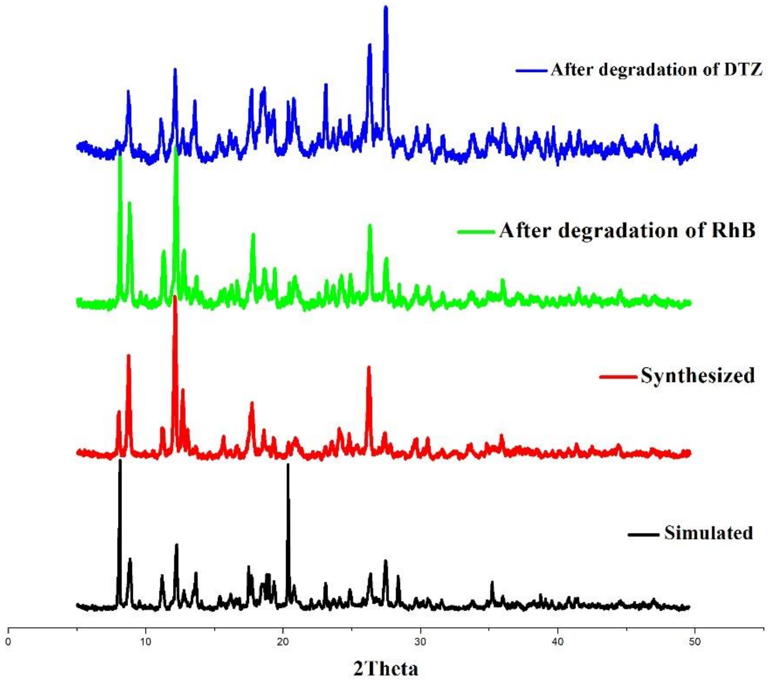

Figure. S6 PXRD plots for CP **3**.

Supporting Information

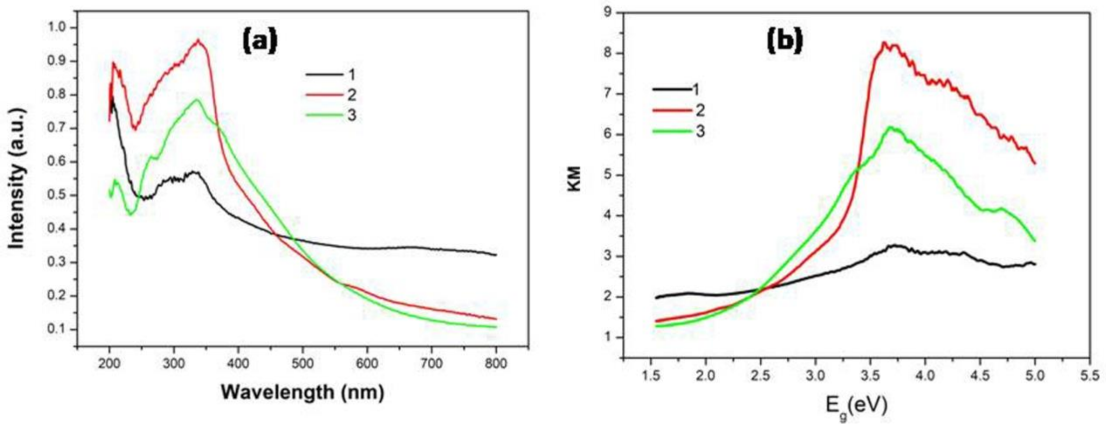

Figure. S7 (a) Solid state UV-Vis for CPs; (b) DRS plots for CPs.

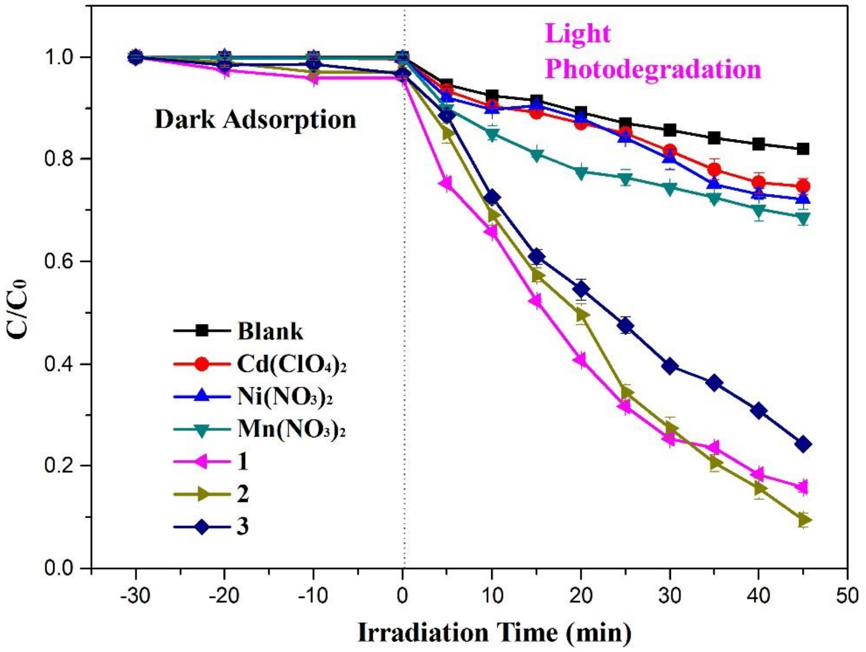

Figure. S8 graph representing plot between C/C_0 vs. irradiation time from different photocatalysis behavior on the DFT molecule.

Supporting Information

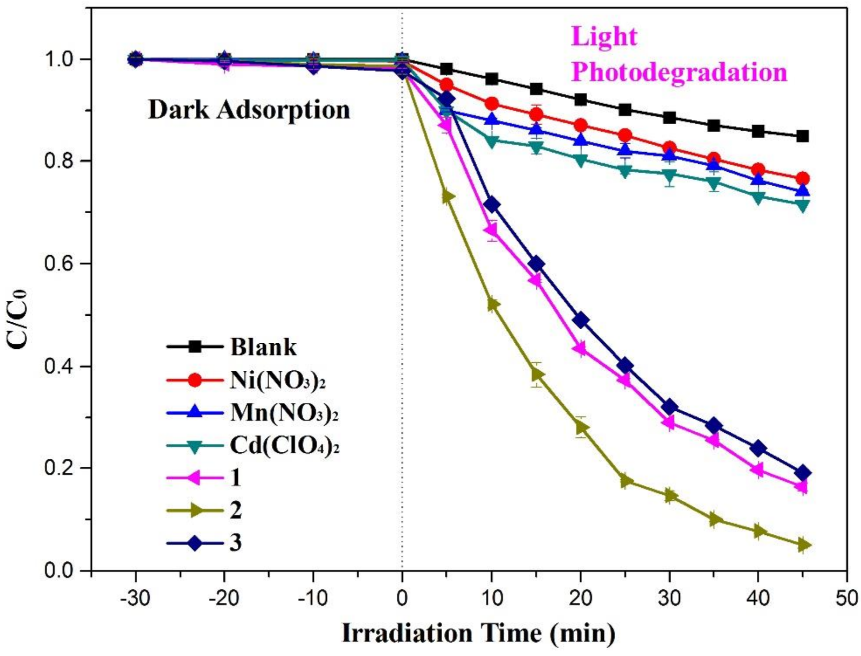

Fig. S9 graph representing plot between C/C_0 vs. irradiation time from different photocatalysis behavior on the Rh B molecule.

Table S1.Selected bond distances (Å) and angles (°) of **1, 2** and **3**

Parameter	**1**	**2**	**3**
Formula	$C_{16}H_{12}N_2NiO_8$	$C_{56}H_{37}Mn_3N_8O_{16}$	$C_{16}H_{12}CdN_2O_8$
Formula weight	453.22	1242.75	472.68
Crystal system	monoclinic	triclinic	monoclinic
Space group	C2/c	P-1	C2/c
Crystal color	green	pink	yellow
a, [Å]	13.9385(10)	11.1841(9)	13.4259(17)
b，[Å]	15.4139(10)	16.4828(14)	15.3909(15)
c，[Å]	16.8615(12)	16.7565(14)	15.619(2)
α，[°]	90	115.6620(10)	90
β，[°]	106.6609(11)	98.114(2)	99.496(14)
γ，[°]	90	105.482(2)	90

Supporting Information

V, Å^3	3470.6(4)	2563.4(4)	3183.2(7)
Z	8	2	8
ρ_{calcd}, g/cm^3	1.735	1.610	1.973
μ, mm^{-1}	1.181	0.812	1.425
F(000)	1864.0	1264.0	1872.0
θRange, deg	2.522-27.649	2.352-26.466	3.530-29.224
Reflection collected	10380	11158	3895
Goodness-of-fit on F^2	1.022	1.065	0.998
R_1, wR_2(I>2σ(I))*	0.0353, 0.0997	0.0655, 0.1620	0.1106, 0.2465
R_1, wR_2(all data)**	0.0442, 0.1048	0.1299, 0.2054	0.2602, 0.3270

*$R=\sum(F_o-F_c)/\sum(F_o)$, **$wR_2=\{\sum[w(F_o^2-F_c^2)^2]/\sum(F_o^2)^2\}^{1/2}$.

Table S2.Selected bond distances (Å) and angles (°) of **1**

Ni(1)-O(1)	1.9841(16)	Ni(1)-O(3A)	1.9838(16)
Ni(1)-O(8)	2.0554(19)	Ni(1)-O(7)	2.0945(19)
Ni(1)-O(6B)	2.0997(15)	Ni(1)-O(5B)	2.1223(16)
O(1)-Ni(1)-O(3A)	104.60(7)	O(1)-Ni(1)-O(8)	88.66(8)
O(3A)-Ni(1)-O(8)	89.64(8)	O(7)- Ni(1)-O(1)	87.57(8)
O(3A)-Ni(1)-O(7)	88.72(8)	O(8)-Ni(1)-O(7)	175.37(7)
O(1)-Ni(1)-O(6B)	158.83(7)	O(3A)-Ni(1)-O(6B)	96.41(6)

Symmetrycodes: A: x, -y, 0.5+z; B: x, 1-y, 0.5+z

Table S3.Selected bond distances (Å) and angles (°) of **2**

Mn(1)-O(1)	2.121(3)	Mn(1)-O(4A)	2.136(4)
Mn(1)-O(11)	2.164(4)	Mn(1)-O(14)	2.181(3)
Mn(1)-O(13)	2.248(4)	Mn(1)-O(9)	2.303(4)
Mn(2)-O(3)	2.089(4)	Mn(2)-O(12B)	2.092(4)
Mn(2)-O(14B)	2.105(4)	Mn(2)-O(4)	2.271(5)
Mn(2)-N(3)	2.287(5)	Mn(3)-O(5C)	2.056(4)
Mn(3)-O(14)	2.069(3)	Mn(3)-O(2)	2.133(4)
Mn(3)-N(1)	2.237(5)	Mn(3)-N(2)	2.275(5)
O(4A)-Mn(1)-O(1)	164.58(15)	O(11)-Mn(1)-O(1)	88.95(16)
O(4A)-Mn(1)-O(14)	95.90(14)	O(13)-Mn(1)-O(1)	81.87(16)
O(4A)-Mn(1)-O(13)	83.10(16)	O(9)-Mn(1)-O(11)	178.14(15)

O(3)-Mn(2)-O(12B)	103.87(16)	O(3)-Mn(2)-N(4)	97.06(15)
O(12B)-Mn(2)-N(4)	154.49(16)	O(3)-Mn(2)-N(3)	121.82(15)
O(12B)-Mn(2)-N(3)	84.08(16)	N(4)-Mn(2)-N(3)	72.45(16)
O(14)-Mn(3)-O(5C)	109.38(16)	O(2)-Mn(3)-O(5C)	90.43(15)
O(2)-Mn(3)-O(14)	104.87(14)	N(1)-Mn(3)-O(5C)	138.54(18)
O(2)-Mn(3)-N(1)	92.49(15)	N(2)-Mn(3)-O(5C)	90.67(17)

Symmetrycodes: A: 1+x, y, z; B: -1+x, y, z; C: 1-x, -y, -z.

Table S4. Selected bond distances (Å) and angles (°) of **3**

Cd(1)-O(1)	2.183(10)	Cd(1)-O(2A)	2.161(9)
Cd(1)-O(3B)	2.375(10)	Cd(1)-O(5C)	2.300(11)
Cd(1)-O(6C)	2.386(10)	Cd(1)-O(7)	2.337(10)
O(3B)-Cd(1)-O(1)	92.6(4)	O(5C)-Cd(1)-O(1)	96.6(4)
O(6C)-Cd(1)-O(1)	151.4(3)	O(7)-Cd(1)-O(1)	85.9(4)
O(2A)-Cd(1)-O(1)	116.2(4)	O(2A)-Cd(1)-O(3B)	93.5(4)
O(2A)-Cd(1)-O(5C)	147.1(4)	O(2A)-Cd(1)-O(7)	90.8(4)

Symmetrycodes: A: 1-x,- y,1- z; B: 1-x, y, 1.5- z; C: 1-x,1 -y, 1-z.

Table S5. Selected hydrogen bond distances (Å) and angles (°) of **1**

Contact D-H···A	Distance (Å)			Angle
	D-H	H···A	D···A	D-H···A(°)
O(4)-H(4)···O(2)	0.82	1.72	2.489(2)	154
O(7)-H(7A)···O(2)	0.85	2.16	2.968(2)	157
O(7)-H(7B)···O(6)	0.85	1.94	2. 779(2)	169
O(8)-H(8A)···N(1)	0.85	2.02	2.840(3)	162
C(7)-H(7)···O(1)	0.93	2.37	2.702(3)	100
C(7)-H(7)···N(1)	0.93	2.52	2.853(3)	102
C(12)-H(12)···O(6)	0.93	2.52	2.833(3)	100

Table S6.Selected hydrogen bond distances (Å) and angles (°) of **2**

Contact D-H···A	Distance (Å)			Angle
	D-H	H···A	D···A	D-H···A (°)
N(5)-H(5)···O(15)	0.86	2.00	2.793(7)	154
N(7)-H(7)···O(6)	0.86	1.89	2.681(7)	152
O(8)-H(8)···N(8)	0.82	1.84	2.603(17)	154
O(13)-H(13)···O(16)	0.95	1.94	2.773(8)	145
O(15)-H(15A)···O(16)	0.85	2.06	2.877(10)	161

O(16)-H(16A)···N(6)	0.90	2.14	2.915(7)	143
O(16)-H(16B)···O(9)	1.00	2.09	3.017(7)	153
O(10)-H(10B)···O(4)	0.82	1.96	2.738(5)	160

Table S7.Selected hydrogen bond distances (Å) and angles (°) of **3**

Contact D-H···A	Distance (Å)			Angle
	D-H	H···A	D···A	D-H···A (°)
N(1)-H(1)···O(8)	0.86	1.87	2.696(17)	159
O(7)-H(7A)···O(4)	0.85	1.89	2.736(12)	169
O(7)-H(7B)···O(6)	0.85	2.07	2.837(13)	149
O(8)-H(8B)···O(5)	0.85	2.29	2.757(16)	115
C(5)-H(5)···O(3)	0.93	2.58	3.381(14)	144
C(7)-H(7)···N(1)	0.93	2.59	2.891(13)	100
O(13)-H(13)···O(6)	0.93	2.56	2.866(14)	100
